# A real‐world study to assess the association of cardiovascular adverse events (CVAEs) with ibrutinib as first‐line (1L) treatment for patients with chronic lymphocytic leukaemia (CLL) in the United States

**DOI:** 10.1002/jha2.638

**Published:** 2023-01-23

**Authors:** Anthony Mato, Boxiong Tang, Soraya Azmi, Keri Yang, Yi Han, Xiaowei Zhang, Lindsey Roeker, Nicola Wallis, Jennifer C. Stern, Eric Hedrick, Jane Huang, Jeff P. Sharman

**Affiliations:** ^1^ Division of Hematological Oncology Memorial Sloan Kettering Cancer Center New York New York USA; ^2^ BeiGene, Ltd. Emeryville California USA; ^3^ BeiGene‐UK, Ltd London UK; ^4^ BeiGene, Ltd. Cambridge Massachusetts USA; ^5^ Willamette Valley Cancer Institute Eugene Oregon USA

**Keywords:** cardiovascular adverse events, CLL, ibrutinib, real‐world evidence

## Abstract

Ibrutinib, a Bruton's tyrosine kinase inhibitor, is often used as first‐line (1L) treatment of chronic lymphocytic leukaemia (CLL); however, it is associated with an increased risk for cardiovascular adverse events (CVAEs). This real‐world study adds to existing literature by simultaneously investigating the correlation between pre‐existing CV risk factors and the relative cardiotoxicity of ibrutinib vs other therapies in CLL/small lymphocytic lymphoma (SLL). Using a real‐world database, the risk of subsequent CVAEs (any CVAE, atrial fibrillation [AF], or hypertension) were compared among patients who received 1L ibrutinib monotherapy or another type of non‐ibrutinib therapy, grouped as intensive (IT) or non‐intensive therapy (NIT). Each patient's baseline CV risk was estimated using the Framingham risk score. Inverse probability treatment weighting was incorporated into a logistic regression model to reduce baseline imbalance. Results showed ibrutinib was significantly associated with higher risk of CVAEs regardless of baseline CV risk. Compared with IT, odds ratios of any CVAE, hypertension, or AF were 2.61, 3.66, and 3.02, respectively vs 1.88, 2.13, and 2.46, respectively, with NIT. Sensitivity analyses confirmed the findings were robust. These results suggest clinical caution should be taken when selecting ibrutinib for patients with CLL/SLL, especially in those with high baseline CV risk.

## 1 INTRODUCTION

Chronic lymphocytic leukaemia (CLL) is the most common leukaemia among adults in Western countries, with an incidence rate of 4–6 cases per 100,000 persons per year [1]. More than 70% of patients diagnosed with CLL are older than 65 years [1], with men more frequently affected than women (1.7:1 ratio) [2]. Small lymphocytic lymphoma (SLL) is considered a different manifestation of the same disease [3]. There have been significant advances in treatment of CLL in recent years with the availability of Bruton tyrosine kinase (BTK) inhibitors, beginning with ibrutinib. This has transformed the therapeutic landscape and resulted in improved patient prognosis and survival. With increased cancer survivorship, cardiovascular disease has emerged as an important cause of non‐malignancy related death in cancer survivors some of which may be related to cardiotoxicities associated with cancer treatments [4, 5]. Recent evidence from the UK Clinical Practice Research Datalink primary care data has shown that survivors of a variety of cancers, including haematologic cancers, have an increased medium‐ to long‐term risk of developing 1 or more cardiovascular diseases (CVDs) compared with the general population [6].

Among patients with CLL, the incidence and prevalence of CVD are high. For example, in a Swedish population‐based study, out of 2,078 patients with CLL, 37% had CVD at the time of start of 1L CLL therapy, and 145 of 521 (28%) patients who did not have prior CVD developed new disease within 5 years of starting 1L therapy. CVD was also reported to be the main cause of 131 of 678 deaths (19%) [7]. Ibrutinib, the first‐generation covalent BTK inhibitor, is commonly used in the treatment of CLL and has been associated with increased rates of cardiovascular adverse events (CVAEs) [8, 9, 10, 11, 12]. In particular, treatment with ibrutinib has been widely reported to be associated with increased risk for new or worsening hypertension and atrial fibrillation/atrial flutter (AF) [8, 9, 12, 13].

With the exception of a 2021 study from Canada [14], earlier published studies using real‐world data from patients with CLL who were treated with ibrutinib have not examined the influence of baseline cardiovascular (CV) risk factors on subsequent ibrutinib‐associated CVAEs. Rather, past studies have mainly focused on the incidence rates of CVAEs related to ibrutinib, or have not performed a comparison of CVAEs related to different treatment regimens [7, 12, 15, 16, 17]. Patients with CLL often have baseline characteristics, including CV comorbidities, that influence a physician's choice of treatment [18]. Furthermore, clinical trials typically restrict the enrollment of patients with significant CV morbidities, limiting the understanding of the relationship between CV morbidities and CVAE risk of ibrutinib treatment in practice [19, 20, 21, 22, 23, 24, 25, 26]. Therefore, it is important for studies of real‐world data to consider a patient's baseline CV risk factors when analysing side effects of different treatment options.

One of the challenges in conducting a real‐world study to compare BTK inhibitor use with other treatments is that when choosing therapy, the treating physician must take into consideration the fitness of the patient as well as disease factors such as cytogenetic mutation status. For instance, a patient with active disease who is fit, with no deletion of 17p (del[17p]) mutation, may be treated differently if the patient was unfit. Hence, the choice of ibrutinib (or another BTK inhibitor/treatment) may, by default, be biased toward a certain type of patient. This includes the level of CV fitness of each patient. These treatment choices made by each physician are then reflected in real‐world data, and as such, any real‐world study must be mindful of the potential confounding effects of other patient factors, including CV comorbidities.

In this study, we investigated the role of pre‐existing CV risk factors and the relative cardiotoxicity of ibrutinib monotherapy (IM) vs other therapies, grouped into intensive therapy (IT) and non‐intensive therapy (NIT). The objective of this study was to ascertain whether ibrutinib confers additional CVAE risk over and above a patient's pre‐existing baseline CV risk, measured in terms of (i) any CVAE, (ii) new or worsening hypertension, and (iii) new or worsening AF.

## 2 METHODS

### 2.1 Study design and data source

This study used the de‐identified electronic health record (EHR)‐derived Flatiron Health database. The Flatiron Health database is a nationwide longitudinal database founded in 2012, comprised of de‐identified patient‐level structured and unstructured data, curated via technology‐enabled abstraction [27, 28]. At the time of abstraction, the de‐identified data originated from approximately 280 US cancer clinics (≈800 sites of care, covering about 35% of US community oncology practices). The majority of patients in the database originate from community oncology settings; relative community/academic proportions may vary depending on study cohort. Data from Flatiron Health undergo a rigorous quality control process prior to use.

**FIGURE 1:**
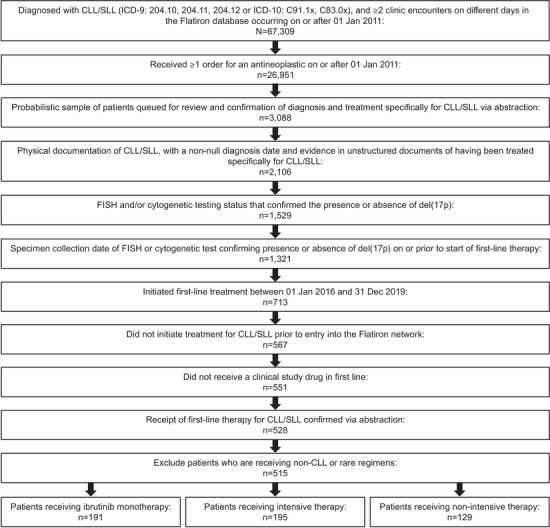
Attrition diagram. CLL, chronic lymphocytic leukaemia; FISH, fluorescence in situ hybridization; SLL, small lymphocytic lymphoma

Selection into the analysis cohort involved the following steps (Figure 1). All patient records that met the following criteria were retrieved: patients aged ≥ 18 years, diagnosis of CLL or SLL (ICD‐9 codes: 204.10, 204.11, 204.12; ICD‐10 codes: C91.1x, C83.0x), ≥ 2 clinic encounters, and initiation of 1L treatment between 1 January 2016, and 31 December 2019. The index date was defined as the date of 1L treatment initiation. Patient data were excluded if treatment for CLL was initiated prior to entry into the Flatiron Health network, the patient received a clinical study drug as 1L treatment, or the patient simultaneously received ibrutinib and infusion medications.

CV risk factors were abstracted from the Flatiron Health EHR based on the presence of a documented CV risk factor prior to the start of 1L treatment. Similarly, CVAEs were abstracted based on documented new occurrence of a CVAE or the worsening of a prior condition. Both new and worsening cases of CVAEs were documented by providers in the patient record. See Supplemental Material page 1–3 for list of abstracted CV risk factors and prior conditions.

Three patient groups were defined based on the 1L treatment: (i) IM; (ii) IT; (iii) NIT (Table 1). IT was defined as therapy that was considered aggressive and less tolerable (most were bendamustine+anti‐CD20 antibody). Bendamustine + rituximab was included in this group based on the frailty of patients that could be prescribed this medication. NIT was defined as therapy that was less aggressive and more tolerable (most were anti‐CD20 antibody monotherapy).

**TABLE 1:** Treatment groups

**Treatment group**	*** n***
**Ibrutinib monotherapy**	191
**Intensive therapy**	195
Bendamustine + anti‐CD20 antibody^a^	142
Cyclophosphamide + fludarabine + anti‐CD20 antibody^a^	42
Cyclophosphamide + vincristine + anti‐CD20 antibody^a^	5
Fludarabine + anti‐CD20 antibody^a^	5
Cyclophosphamide + doxorubicin + vincristine + anti‐CD20 antibody^a^	1
**Non‐Intensive therapy**	129
Anti‐CD20 antibody^a^	79
Chlorambucil + anti‐CD20 antibody^a^	36
Chlorambucil	8
Venetoclax + anti‐CD20 antibody^a^	5
Cyclophosphamide + anti‐CD20 antibody^a^	1
**All patients**	515

^a^ Anti‐CD20 antibody includes ofatumumab, rituximab, and obinutuzumab.

### 2.2 Independent variables and outcome variables

Patient characteristics used as baseline variables were age, body mass index (BMI), systolic blood pressure (SBP), smoking status, diabetes status, Rai stage at diagnosis, Eastern Cooperative Oncology Group performance status (ECOG PS) at index date, del(17p) status, immunoglobulin heavy chain variable region (*IgHV*) mutation status, history of acute coronary syndrome (ACS)/myocardial infarction (MI), angina/coronary revascularization, congestive heart failure, AF, other arrhythmias, cerebrovascular disease, peripheral arterial disease, and hypercholesterolemia.

The primary study outcomes were the occurrence of (i) any CVAE, (ii) new or worsening hypertension, and (iii) new or worsening AF. The outcome of any CVAE included categories (ii) and (iii) as well as any other CVAEs captured from the patient records including ≥ 1 events of ACS (ie, new MI; chest pain, cardiac; angina), new or worsening AF, new or worsening heart failure (ie, congestive heart failure, cardiomyopathy, rheumatic heart failure, other heart failure listed by Flatiron Health), new or worsening hypertension, or new or worsening other arrhythmias (ie, bradycardia, arrhythmia not otherwise specified).

### 2.3 Statistical analysis

Three main analysis steps were performed. In the first step, to characterize pre‐treatment CV risks, the Framingham risk score (FRS) was calculated, which included age, sex, BMI, SBP, smoking status, and diabetes status, as a continuous variable [29]. The FRS was then used as an independent variable in univariate logistic regression against the CVAE endpoints of any CVAE, new or worsening hypertension, and new or worsening AF. Other baseline variables not used in the FRS calculation were treated as independent covariates. Descriptive analysis was performed for all variables by treatment cohort. For continuous variables, means and SDs were reported; for categorical variables, frequencies and counts were reported. Statistical tests were performed on each variable to check for any significant imbalance across the treatment cohorts. If the continuous variable was normally distributed, the t‐test was applied; otherwise, the signed‐rank test was used. The chi‐square test was applied for categorical variables and the Fisher's exact test was applied if at least 1 cell had an expected frequency less than 5.

Secondly, logistic regression with inverse probability treatment weighting (IPTW) was used to investigate the main effects of baseline CV risk based on the FRS and 1L treatment on CVAE outcomes while controlling for other potential baseline confounders [30]. Potential confounders included in the analysis were Rai stage at diagnosis, ECOG PS at index date, del(17p) status, *IgHV* mutation status, history of ACS/MI, angina/coronary revascularization, congestive heart failure, AF, other arrhythmias, cerebrovascular disease, peripheral arterial disease, and hypercholesterolemia. Because IPTW allows for comparisons between only 2 groups at a time, IM was compared against IT and NIT separately.

Finally, 3 types of sensitivity analyses were performed to evaluate the robustness of the findings from the logistic regression analyses described above. First, to check the result stability against IPTW method variation, stepwise variable selection was used to derive a parsimonious version of IPTW (details of this sensitivity analysis are in the Supplemental Material page 3). Second, to evaluate robustness against treatment grouping, the IT and NIT cohorts were combined and compared with the IM cohort. Third, to address confounding between treatment choice and FRS, the interaction term of treatment choice and FRS was added to the model.

## 3 RESULTS

### 3.1 Baseline characteristics

A total of 515 patients met the eligibility requirements following the selection process (Figure 1). Of these, 191 patients received IM, 195 received IT, and 129 received NIT (Table 1). The most common treatment regimens in the IT group were bendamustine plus anti‐CD20 therapy and cyclophosphamide plus fludarabine plus anti‐CD20 therapy. The most common treatment regimens in the NIT group were anti‐CD20 monotherapy and chlorambucil plus anti‐CD20 therapy.

Patients in the IM group were significantly older than those in the IT group (mean [SD], 71.2 [9.9] years vs 66.2 [10.6] years; *P* < .05) but significantly younger than those in the NIT group (74.5 [7.9]; *P* < .05; Table 2). Of the patients included in this analysis, most in all 3 groups had documented ECOG PS of 0 at the index date, where data were available (IM: 30.9% and 28.8%; IT: 22.6% and 43.1%; NIT: 26.4% and 35.7%). The presence of del(17p) was significantly greater in the IM group than in the IT and NIT groups (26.7%, 3.6%, 3.1%, respectively; *P* < .05). The median FRSs were 0.340, 0.299, and 0.419 for the IM, IT, and NIT groups, respectively; an FRS of 0.2 (20%) is considered a high CV risk [21].

**TABLE 2:** Baseline characteristics

**Baseline characteristic**	**IM** **(*n* = 191)**	**IT** **(*n* = 195)**	**NIT** **(*n* = 129)**
**Sex, male, *n* (%)**	120 (62.8)	136 (69.7)	68 (52.7)
**Age at index date, mean (SD), y**	71.2 (9.9)	66.2 (10.6)^a^	74.5 (7.9)^b^
**Rai stage at diagnosis, *n* (%)**			
**0**	59 (30.9)	44 (22.6)	34 (26.4)
**I**	33 (17.3)	26 (13.3)	13 (10.1)
**II**	9 (4.7)	14 (7.2)	5 (3.9)
**III**	16 (8.4)	14 (7.2)	7 (5.4)
**IV**	15 (7.9)	31 (15.9)	11 (8.5)
**Not documented**	59 (30.9)	66 (33.9)	59 (45.7)
**Eastern Cooperative Oncology Group performance status (ECOG PS) at index date, n (%)**			
**0**	55 (28.8)	84 (43.1)^a^	46 (35.7)
**1**	51 (26.7)	46 (23.6)^a^	41 (31.8)
**2**	15 (7.9)	6 (3.1)^a^	8 (6.2)
**3**	2 (1.1)	1 (0.5)^a^	1 (0.8)
**4**	1 (0.5)	2 (1.0)^a^	1 (0.8)
**Unknown/not documented**	67 (35.1)	56 (28.7)^a^	32 (24.8)
**del(17p) present, *n* (%)**	51 (26.7)	7 (3.6)^a^	4 (3.1)^b^
***IgHV* mutated, *n* (%)**	29 (15.2)	35 (18.0)	26 (20.2)
**Body mass index at baseline, mean (SD), kg/m^2^**	28.5 (6.5)	29.0 (6.4)	28.5 (6.7)
**Framingham risk score, median**	0.340	0.299	0.419
**Diabetes mellitus, *n* (%)**	107 (56.0)	86 (44.1)^a^	82 (63.6)
**Smoking status, yes, *n* (%)**	48 (25.1)	47 (24.1)	36 (27.9)
**Acute coronary syndrome/myocardial infarction, *n* (%)**	15 (7.9)	13 (6.7)	15 (11.6)
**Angina/coronary revascularization, *n* (%)**	5 (2.6)	4 (2.1)	11 (8.5)^b^
**Congestive heart failure, *n* (%)**	10 (5.2)	4 (2.1)	10 (7.8)
**Pre‐existing hypertension, *n* (%)**	6 (3.1)	2 (1.0)	7 (5.4)
**Atrial fibrillation/atrial flutter, *n* (%)**	27 (14.1)	23 (11.8)	34 (26.4)^b^
**Other arrhythmias, *n* (%)**	97 (50.8)	107 (54.9)	65 (50.4)
**Cerebrovascular disease, *n* (%)**	17 (8.9)	23 (11.8)	26 (20.2)^b^
**Peripheral arterial disease, *n* (%)**	18 (9.4)	9 (4.6)	7 (5.4)
**Hypercholesterolemia, *n* (%)**	131 (68.6)	113 (58.0)^a^	95 (73.6)

^a^ *P* < .05, IM vs IT.

^b^ *P* < .05, IM vs NIT.

*IgHV*, immunoglobulin heavy chain variable region.

### 3.2 CV risk assessments

**FIGURE 2:**
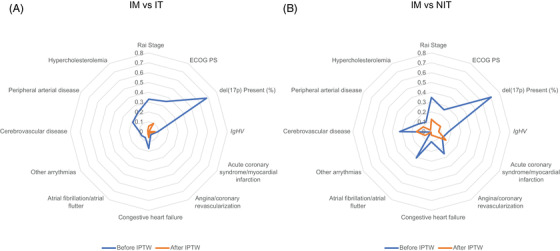
Standard mean difference after inverse probability treatment weighting adjustment for ibrutinib monotherapy (IM) vs (A) intensive therapy (IT) and (B) non‐intensive therapy (NIT). ECOG PS, Eastern Cooperative Oncology Group performance status; *IgHV*, immunoglobulin heavy chain variable region

Univariate logistic regression analysis assessing the impact of baseline CV risk alone, as measured by the FRS, showed that baseline CV risk was associated with CVAE and new or worsening hypertension (Table 3). The IPTW adjustment performed for both IM vs IT and IM vs NIT resulted in improved balance in terms of patient baseline characteristics and comparability (Figure 2 **A,B**).

**TABLE 3:** Univariate logistic regressions of CVAE outcomes against Framingham risk score as an independent variable

**Dependent variable**	**Odds ratio (95% CI)**
**Any CVAE** **^a^**	1.37 (1.13, 1.66)^b^
**New/worsening hypertension**	1.37 (1.07, 1.74)^b^
**New/worsening atrial fibrillation/atrial flutter**	1.16 (0.85, 1.60)

Abbrevitions: ACS, acute coronary syndrome; AF, atrial fibrillation; CVAE, cardiovascular adverse event; MI, myocardial infarction.

^a^ Includes ≥ 1 events of ACS (ie, new MI; chest pain, cardiac; angina), new or worsening AF, new or worsening heart failure (ie, congestive heart failure, cardiomyopathy, rheumatic heart failure, other heart failure listed by Flatiron Health), new or worsening hypertension, or new or worsening other arrhythmias (ie, bradycardia, arrhythmia not otherwise specified).

^b^ *P* < .05.

Following IPTW, logistic regression results showed that both baseline CV risk based on FRS, and IM treatment, significantly and independently predicted occurrence of CVAEs. Main effect analysis showed that compared with IT and NIT, 1L IM treatment was significantly associated with increased risk of CVAEs for all patients. This was true at any FRS level (Table 4). Odds ratios of any CVAEs with IM vs IT and NIT were 2.61 (95% CI: 1.86, 3.67; *P* < .05) and 1.88 (95% CI: 1.32, 2.67; *P* < .05), respectively; for new or worsening hypertension with IM vs IT and NIT, they were 3.66 (95% CI: 2.30, 5.80; *P* < .05) and 2.13 (95% CI: 1.37, 3.31; *P* < .05), respectively; and for new or worsening AF with IM vs IT and NIT, they were 3.02 (95% CI: 1.64, 5.56; *P* < .05) and 2.46 (95% CI: 1.36, 4.44; *P* < .05), respectively.

**TABLE 4:** Analysis results from logistic regression with inverse probability treatment weighting

**Groups**	**Dependent variable**	**Independent variable**	**Odds ratio (95% CI)**
**Ibrutinib monotherapy vs intensive therapy**	Any CVAEs	1L treatment	2.61 (1.86, 3.67)^a^
		Framingham risk score	1.48 (1.24, 1.75)^a^
	New or worsening hypertension	1L treatment	3.66 (2.30, 5.80)^a^
		Framingham risk score	1.27 (1.03, 1.57)^a^
	New or worsening AF	1L treatment	3.02 (1.64, 5.56)^a^
		Framingham risk score	1.20 (0.91, 1.58)
**Ibrutinib monotherapy vs non‐intensive therapy**	Any CVAEs	1L treatment	1.88 (1.32, 2.67)^a^
		Framingham risk score	1.39 (1.17, 1.65)^a^
	New or worsening hypertension	1L treatment	2.13 (1.37, 3.31)^a^
		Framingham risk score	1.36 (1.11, 1.68)^a^
	New or worsening AF	1L treatment	2.46 (1.36, 4.44)^a^
		Framingham risk score	1.51 (1.16, 1.96)^a^

Abbreviations: 1L, first‐line; AF, atrial fibrillation/atrial flutter; CVAE, cardiovascular adverse event.

^a^ *P*  < .05.

### 3.3 Sensitivity analyses

Sensitivity analyses confirmed that the findings were robust against changes in IPTW methodology, treatment grouping changes, and model specification (Tables 5, 6, 7).

**TABLE 5:** Sensitivity analysis 1: IPTW stepwise variable selection model

**A. IM vs IT**					
**Dependent variable**	**Independent variable**	**Beta coefficients**	**Standard error**	**Odds ratio (95% CI)**	***P*‐value**
Any CVAEs	1L treatment	0.47	0.087	2.56 (1.83, 3.60)	**<.001**
	FRS	0.38	0.088	1.47 (1.23, 1.74)	**<.001**
New or worsening hypertension	1L treatment	0.65	0.118	3.63 (2.29, 5.76)	**<.001**
	FRS	0.18	0.108	1.20 (0.97, 1.48)	.093
New or worsening AF	1L treatment	0.49	0.150	2.65 (1.47, 4.77)	**.001**
	FRS	0.21	0.139	1.23 (0.94, 1.61)	.138

**B. IM vs NIT**					
**Dependent variable**	**Independent variable**	**Beta coefficients**	**Standard error**	**Odds ratio (95% CI)**	***P*‐value**
Any CVAEs	1L treatment	0.31	0.089	1.85 (1.31, 2.62)	**<.001**
	FRS	0.30	0.086	1.35 (1.14, 1.60)	**<.001**
New or worsening hypertension	1L treatment	0.42	0.118	2.33 (1.47, 3.70)	**<.001**
	FRS	0.27	0.109	1.31 (1.06, 1.62)	**.014**
New or worsening AF	1L treatment	0.43	0.148	2.39 (1.34, 4.25)	**.003**
	FRS	0.47	0.133	1.60 (1.23, 2.08)	**<.001**

*Note*: *P*‐values <.05 were considered significant and are in bold.

Abbreviations: 1L, first‐line; AF, atrial fibrillation/atrial flutter; CVAE, cardiovascular adverse event; FRS, Framingham risk score; IM, ibrutinib monotherapy; IPTW, inverse probability treatment weighting; IT, intensive therapy; NIT, non‐intensive therapy.

**TABLE 6:** Sensitivity analysis 2: Combining comparison groups (IT + NIT) vs IM

**A. IPTW using non‐parsimonious propensity score model** **^a^**					
**Dependent variable**	**Independent variable**	**Beta coefficients**	**Standard error**	**Odds ratio (95% CI)**	***P*‐value**
Any CVAEs	1L treatment	0.40	0.072	2.24 (1.69, 2.97)	**<.001**
	FRS	0.37	0.071	1.44 (1.26, 1.66)	**<.001**
New or worsening hypertension	1L treatment	0.53	0.094	2.87 (1.99, 4.15)	**<.001**
	FRS	0.28	0.087	1.33 (1.12, 1.58)	**.001**
New or worsening AF	1L treatment	0.47	0.124	2.56 (1.58, 4.16)	**<.001**
	FRS	0.29	0.112	1.34 (1.07, 1.66)	**.010**

**B. IPTW using variable selection method** **^b^**					
**Dependent variable**	**Independent variable**	**Beta coefficients**	**Standard error**	**Odds ratio (95% CI)**	***P*‐value**
Any CVAEs	1L treatment	0.40	0.071	2.23 (1.68, 2.95)	**<.001**
	FRS	0.35	0.071	1.42 (1.23, 1.63)	**<.001**
New or worsening hypertension	1L treatment	0.52	0.094	2.83 (1.96, 4.09)	**<.001**
	FRS	0.22	0.087	1.24 (1.05, 1.47)	**.013**
New or worsening AF	1L treatment	0.44	0.122	2.39 (1.48, 3.86)	**<.001**
	FRS	0.28	0.112	1.33 (1.07, 1.65)	**.011**

*Note*: *P*‐values <.05 were considered significant and are in bold.

Abbreviations: 1L, first‐line; AF, atrial fibrillation/atrial flutter; CVAE, cardiovascular adverse event; FRS, Framingham risk score; IM, ibrutinib monotherapy; IPTW, inverse probability treatment weighting; IT, intensive therapy; NIT, non‐intensive therapy.

^a^ Method used was the same as in the main analysis but the IT and NIT group data are combined.

^b^ Method used was the same as in Table 5 but the IT and NIT group data are combined.

**TABLE 7:** Sensitivity analysis 3: Interaction effects between treatment and baseline cardiovascular risk

**A. IM vs IT**				
**Dependent variable**	**Independent variable**	**Beta coefficients**	**Standard error**	***P*‐value**
**Any CVAEs**	1L treatment	0.48	0.088	**<.001**
	FRS	0.33	0.093	**<.001**
	1L treatment × FRS	0.24	0.093	**.010**
**New or worsening hypertension**	1L treatment	0.65	0.120	**<.001**
	FRS	0.17	0.125	.171
	1L treatment × FRS	0.14	0.125	.261
**New or worsening AF**	1L treatment	0.59	0.169	**.001**
	FRS	0.02	0.178	.918
	1L treatment × FRS	0.30	0.178	.091

**B. IM vs NIT**				
**Dependent variable**	**Independent variable**	**Beta coefficients**	**Standard error**	***P*‐value**
**Any CVAEs**	1L treatment	0.25	0.091	**.005**
	FRS	0.32	0.090	**<.001**
	1L treatment × FRS	0.29	0.090	**.001**
**New or worsening hypertension**	1L treatment	0.37	0.118	**.002**
	FRS	0.31	0.107	**.004**
	1L treatment × FRS	0.01	0.107	.904
**New or worsening AF**	1L treatment	0.45	0.166	**.007**
	FRS	0.41	0.140	**.004**
	1L treatment × FRS	0.00	0.140	.987

*Note*: *P*‐values < .05 were considered significant and are in bold.

Abbreviations: 1L, first‐line; AF, atrial fibrillation/atrial flutter; CVAE, cardiovascular adverse event; FRS, Framingham risk score; IM, ibrutinib monotherapy; IT, intensive therapy; NIT, non‐intensive therapy.

## 4 DISCUSSION

Past studies have shown that ibrutinib is associated with higher rates of AF and hypertension, but how these events may have been influenced by patients’ baseline CV risk had not been explored. Comparison with other 1L treatment regimens was also lacking. In the results of this real‐world evidence study, underlying CV risk factors were shown to correlate with subsequent CVAEs, but baseline CV risk did not account for all risk of CVAEs among patients who received ibrutinib monotherapy. Treatment with ibrutinib monotherapy, therefore, accounts for a higher level of CV risk over and above that of any underlying cardiac conditions.

A 2021 study by Abdel‐Qadir et al. used data from the Ontario Health Insurance Plan and other sources of real‐world data to investigate CV risk in patients treated with ibrutinib [14]. The study compared patients treated with ibrutinib with ibrutinib‐unexposed patients by age, pre‐existing AF, anticoagulant use, and propensity score and showed that ibrutinib was associated with an approximately 2‐fold higher risk of AF, bleeding, and heart failure, but not of acute MI or stroke. Although the study by Abdel‐Qadir et al. used a larger dataset than the current study, it did not investigate the effects of ibrutinib treatment on hypertension, nor did it consider certain baseline variables that are clinically important in CLL, such as the status of del(17p), *IgHV* mutation status, and ECOG PS [2].

A study by Dickerson et al. (2019) [9] analysed data from patients with CLL treated with ibrutinib in the United States and showed that among patients who developed hypertension, there was an increase in major CV events. The study did not include a direct comparison with other CLL treatments. Separately, a study by Olszewski et al. (2019) [15] used the US SEER‐Medicare registry data to examine treatment patterns and outcomes in patients with CLL treated with ibrutinib, chemotherapy, and/or immunotherapy. Results showed that incidence of AF was significantly higher among patients receiving ibrutinib compared with other treatment types. A study by Baptiste et al. (2019) [11] determined the incidence and predictors of ibrutinib‐related AF. The study included only patients treated with ibrutinib and found that the incidence of AF was 15‐fold higher than in the general population.

A 2021 retrospective chart review from a single institution investigated whether patients with pre‐existing CVD had a higher likelihood of developing new AF than those with no prior CVD, following treatment with ibrutinib [31]. It was found that patients with prior CVD were 2.91 times more likely to develop AF than those without prior CVD, although this study was limited by its small sample size, and the sample of patients came from a single institution. In a study using EHRs from multiple hospitals in Denmark, Aarup et al. (2020) [16] investigated outcomes and adverse events (AEs) in patients with CLL treated with ibrutinib. They reported that any‐grade AF occurred in 15.6% of patients and was the most common AE leading to treatment discontinuation. In summary, although past findings support the observation that ibrutinib is associated with CVAEs (AF in particular), past studies either did not compare the effects of ibrutinib treatment with other treatment options or did not address the impact of underlying CV risk.

Taken together, the current study and prior studies all point to important cardiac considerations that should be weighed when considering ibrutinib use. Current guidelines for the treatment of CLL indicate that the second generation BTK inhibitor, acalabrutinib (approved for the treatment of CLL), and the next generation BTK inhibitor, zanubrutinib (not approved for CLL), are both among the preferred 1L treatment options in addition to ibrutinib [32]. Both compounds are being evaluated against ibrutinib in ongoing Phase 3 trials [20, 22] and both have randomized prospective data demonstrating more favourable CV safety profiles compared with ibrutinib. Initial safety results from the acalabrutinib study showed that rates of AF and hypertension were greater with ibrutinib (AF: 16.0% vs 9.4%; hypertension: 23.2% vs 9.4%) [20]. Results from the first interim analysis of the ALPINE study showed that the rate of AF was significantly lower with zanubrutinib than ibrutinib (2.5% vs 10.1%; 2‐sided *P* = .0014) [22]. These initial safety results are cautiously encouraging, although long‐term data are needed.

There were several limitations in the current study that should be considered. Because this study used real‐world data, there is a possibility of inaccurate or incomplete information, such as the relatively high number of missing ECOG PS and Rai stage values. Additionally, a manual data abstraction process was used, and hence there may have been under‐reporting of AE cases. Furthermore, the data originated from oncology practice settings rather than primary care or cardiology specialties, which further increases the possibility of under‐capturing CVAEs. Although it could be conceived that the grouping of treatments as IT and NIT may not be warranted, this concern was addressed by combining IT and NIT groups in a sensitivity analysis. Despite this, the results remained robust and were similar to the separate group analyses. While the FRS does not take into consideration all possible comorbidities, it has been shown to be an effective predictor of both individual CV events and general CV risk and can be considered a good representation of CV comorbidities [29]. Additionally, when using real‐world data, it can be harder to prevent bias by balancing treatment groups [33]. IPTW was used in this study in an attempt to balance patients in terms of CV fitness and other clinical characteristics. Finally, while other BTK inhibitors were available for treatment at the time of writing, there were not enough patients treated with them in this dataset to conduct the same analysis, so only ibrutinib was investigated in this study. In the future, it will be useful to conduct similar studies of CV toxicity with other BTK inhibitors.

In conclusion, this real‐world study showed that even when underlying CV risk was taken into account, patients with CLL treated with 1L IM had significantly higher risk of CVAEs, including hypertension and AF, compared with other IT and NIT regimens. These findings suggest that clinical caution should be applied in using ibrutinib treatment for patients with CLL/SLL, especially those who are at an elevated risk for CVD.

## AUTHOR CONTRIBUTIONS

All authors discussed the results and contributed to the final manuscript. All authors had access to the data and controlled the decision to publish. Anthony Mato provided advice to study analysis, reviewed the study results and manuscript. Boxiong Tang contributed to study design, reviewed the study results and manuscript. Soraya Azmi contributed to the design and analysis of the study, managed the study, proposed statistical analysis, reviewed study results, and led manuscript development. Keri Yang contributed to study design, reviewed the study results and manuscript. Yi Han led and advised statistical analysis, reviewed the study results, and reviewed manuscript. Xiaowei Zhang performed statistical analysis and reviewed manuscript. Lindsey Roeker reviewed the study results and manuscript. Nicola Wallis contributed to study design, reviewed the study results and manuscript. Jennifer Stern contributed to study design, reviewed the study results, reviewed manuscript. Eric Hedrick contributed to the concept and design of the study, reviewed the study results and manuscript. Jane Huang contributed to the concept and design of the study, reviewed the study results and manuscript. Jeff Sharman provided advice to study analysis, reviewed the study results and manuscript.

## CONFLICT OF INTEREST

Anthony Mato has been a consultant for AbbVie, Adaptive, AstraZeneca, Celgene Corporation (including data safety monitoring board), Genentech, Johnson & Johnson, LOXO, Regeneron, Pharmacyclics, Sunesis, and TG Therapeutics (including data safety monitoring board); and has received research funding from AbbVie, Acerta, Adaptive, DTRM, Genentech, Johnson & Johnson, Regeneron, LOXO, Pharmacyclics, Portola, and TG Therapeutics. Boxiong Tang, Soraya Azmi, Keri Yang, Yi Han, Xiaowei Zhang, Nicola Wallis, and Jennifer C. Stern are all employees of BeiGene, Inc. Eric Hedrick and Jane Huang were employees of BeiGene, Inc. at the time this study was conducted. Jeff P. Sharman has received research funding from AbbVie, Acerta Pharma, BeiGene, Gilead, Pharmacyclics, Sunesis, and TG Therapeutics; and has served as a consultant to AbbVie, Acerta Pharma, BeiGene, Pharmacyclics, and TG Therapeutics. Lindsey Roeker has been a consultant for AbbVie, Ascentage, AstraZeneca, BeiGene, Janssen, Loxo Oncology, Pharmacyclics, Pfizer, and TG Therapeutics; has equity ownership in Abbott Laboratories; and has received research funding from Aptose Biosciences, Loxo Oncology, Pfizer, and Qilu Puget Sound Biotherapeutics.

## Supporting information

Supplementary material

## Data Availability

All authors had access to the original data for the analyses described here. Upon request and subject to certain criteria, conditions, and exceptions, BeiGene will provide access to data. Data requests may be submitted to DataDisclosure@beigene.com. Funding for this study was provided by BeiGene, Inc.
