# Supplementary material for: A real‐world study to assess the association of cardiovascular adverse events (CVAEs) with ibrutinib as first‐line (1L) treatment for patients with chronic lymphocytic leukaemia (CLL) in the United States
Source: EJHaem. 2023 Jan 23;4(1):135–44. doi: 10.1002/jha2.638 (PMC9928661; doi:10.1002/jha2.638)
Supplement: Supplementary file 1 — Supplementary material [file JHA2-4-135-s001.docx]

**Title: A real-world study to assess the association of cardiovascular adverse events (CVAEs) with ibrutinib as first-line (1L) treatment for patients with chronic lymphocytic leukemia (CLL) in the United States**

**Authors:** Anthony Mato^1^; Boxiong Tang^2^; Soraya Azmi^2^; Keri Yang^2^; Yi Han^2^; Xiaowei Zhang^2^; Lindsey Roeker^1^; Nicola Wallis^3^; Jennifer C. Stern^4^; Eric Hedrick^4^; Jane Huang^3^; Jeff P. Sharman^5^

^1^Memorial Sloan Kettering Cancer Center, New York, NY, USA; ^2^BeiGene, Ltd., Emeryville, CA, USA; ^3^BeiGene-UK, Ltd, London; ^4^BeiGene, Ltd., Cambridge, MA, USA; ^5^Willamette Valley Cancer Institute, Eugene, OR, USA

**Methods supplemental material**

*Cardiovascular risk factor abstraction*

The following cardiovascular (CV) risk factors were abstracted from Flatiron Health de-identified electronic health record (EHR)-derived data, followed by the terms included under each category:

- Myocardial infarction (MI): acute MI, non–ST-elevation MI (NSTEMI), ST-elevation MI (STEMI), heart attack
- Acute coronary syndrome (ACS): myocardial ischemia, unstable angina
- Angina: angina not otherwise specified (NOS), angina pectoris, stable angina
- Coronary revascularization: coronary artery bypass graft, percutaneous coronary intervention, cardiac stents, coronary stents
- Cerebrovascular disease: stroke, transient ischemic attack, transient cerebral ischemia, cerebrovascular accident, cerebral infarction, cerebral thrombosis, cerebral embolism, cerebral/subarachnoid/subdural hemorrhage, cerebral artery occlusion, cerebral aneurysm
- Peripheral arterial disease: intermittent claudication, history of lower extremity arterial bypass graft, history of lower extremity stent, history of lower extremity angioplasty
- Hypertension: high blood pressure, elevated blood pressure, essential hypertension, primary hypertension
- Hypercholesterolemia: high cholesterol, elevated cholesterol, hyperlipidemia, hypertriglyceridemia, dyslipidemia
- Diabetes: type 1 diabetes mellitus, type 2 diabetes mellitus, insulin-dependent diabetes, non–insulin-dependent diabetes
- Smoking
- Bradycardia: sinus bradycardia, junctional bradycardia, slow heart rate
- Congestive heart failure: cardiac failure, heart failure, ventricular failure, right heart failure, rheumatic heart failure, cardiomyopathy (eg, ischemic, dilated), systolic heart failure, chronic diastolic heart failure
- Atrial fibrillation
- Atrial flutter
- Arrhythmia, other

*CV adverse event abstraction*

The following cardiovascular adverse events (CVAEs) were abstracted from Flatiron Health de-identified EHR-derived data, followed by the terms included under each category:

- ACS: myocardial ischemia, unstable angina
- Atrial fibrillation
- Atrial flutter
- Arrhythmia, other: arrhythmia, cardiac arrhythmia, atrial tachycardia, ventricular tachycardia, supraventricular tachycardia, bundle branch, bundle branch block, bundle branch block right, premature ventricular contractions, supraventricular arrhythmia, supraventricular extrasystoles, atrioventricular block complete, atrioventricular block first degree, ventricular arrhythmia, ventricular fibrillation, tachy-brady syndrome, sick sinus syndrome
- Bradycardia: sinus bradycardia, junctional bradycardia, slow heart rate
- Congestive heart failure: cardiac failure, heart failure, ventricular failure, right heart failure, rheumatic heart failure, cardiomyopathy (ischemic or dilated), systolic heart failure, chronic diastolic heart failure
- Chest pain, cardiac: angina, angina pectoris, chest pain, NOS, chest tightness
- Hypertension: high blood pressure, elevated blood pressure, essential hypertension, primary hypertension
- MI: acute MI, NSTEMI, STEMI, heart attack

**Regression analysis with IPTW**

Regression analysis with inverse probability treatment weighting (IPTW) was performed to adjust for all baseline characteristics excluding age, gender, diabetes status, body mass index, smoking status, systolic blood pressure, and hypertension status. Specifically, the variables used for IPTW were Rai stage at diagnosis, ECOG PS at index date, deletion of 17p (del[17p]) status, immunoglobulin heavy chain variable region (*IgHV*) mutation status, ACS/MI, angina/coronary revascularization, congestive heart failure, atrial fibrillation/atrial flutter, other arrhythmias, cerebrovascular disease, peripheral arterial disease, and hypercholesterolemia.

*Sensitivity analysis 1*

This sensitivity analysis used a **propensity score model with variable selection** which used 2 groups of variables: (i) clinically important potential CVAE confounders: ACS/MI, angina/coronary revascularization, congestive heart failure, atrial fibrillation/atrial flutter, other arrhythmias, cerebrovascular disease, peripheral arterial disease, and hypercholesterolemia; and (ii) variables selected by stepwise regression. After incorporating all the clinically important potential CVAE confounders, stepwise regression was used to select additional variables into the propensity score model. These additional variables subject to selection were Rai stage at diagnosis, ECOG PS at index date, del(17p) status, and *IgHV* mutation status. Statistical selection criterion was *P*≤.1.
